# Anti-glomerular basement membrane glomerulonephritis and thrombotic microangiopathy in first degree relatives; a case report

**DOI:** 10.1186/1471-2369-13-64

**Published:** 2012-07-26

**Authors:** Thomas Idorn, Lone Schejbel, Casper Rydahl, James Goya Heaf, Karen Riis Jølvig, Marie Bergstrøm, Peter Garred, Anne-Lise Kamper

**Affiliations:** 1 Department of Nephrology, Rigshospitalet, University of Copenhagen, Blegdamsvej 9, DK-2100, Copenhagen Ø, Denmark; 2 Department of Clinical Immunology, Rigshospitalet, University of Copenhagen, Blegdamsvej 9, DK-2100, Copenhagen Ø, Denmark; 3 Department of Nephrology, Herlev Hospital, University of Copenhagen, Herlev Ringvej 75, DK-2730, Herlev, Denmark

**Keywords:** Aetiology, Anti-glomerular basement membrane glomerulonephritis, Atypical haemolytic-uremic syndrome, Thrombotic microangiopathy

## Abstract

**Background:**

Anti-glomerular basement membrane glomerulonephritis and thrombotic microangiopathy are rare diseases with no known coherence.

**Case Presentation:**

A daughter and her biological mother were diagnosed with pregnancy-induced thrombotic microangiopathy and anti-glomerular basement membrane glomerulonephritis, respectively. Both developed end-stage renal disease. Exploration of a common aetiology included analyses of HLA genotypes, functional and genetic aspects of the complement system, ADAMTS13 activity and screening for autoantibodies.

The daughter was heterozygous carrier of the complement factor I G261D mutation, previously described in patients with membranoproliferative glomerulonephritis and atypical haemolytic uremic syndrome. The mother was non-carrier of this mutation. They shared the disease associated complement factor H silent polymorphism Q672Q (79602A>G).

**Conclusion:**

An unequivocal functional or molecular association between these two family cases was not found suggesting that the patients probably share another, so far undiagnosed and unknown, predisposing factor. It seems highly unlikely that two infrequent immunologic diseases would occur by unrelated pathophysiological mechanisms within first degree relatives.

## Background

Goodpasture’s disease or anti-glomerular basement membrane glomerulonephritis (anti-GBMGN) has an incidence around one/million/year [1]. Autoantibodies are directed against epitope(s) in the glomerular basement membrane, in response to unknown stimuli [2]. It occurs in siblings and twins and a strong association with the major histocompatibility complex class II gene HLA DR2, different HLA DRB1 genes, HLA DR15 and DR4 has been reported, while DR1 and DR7 seem to protect [3,4].

Thrombotic microangiopathy (TMA) is characterized by haemolytic anaemia, thrombocytopenia and organ injury due to platelet thrombosis in the microcirculation. Depending on predominantly kidney or CNS location, it is classified as haemolytic-uremic syndrome (HUS) or thrombotic thrombocytopenic purpura (TTP). HUS may be typical HUS or atypical HUS (aHUS) including the pregnancy induced form. The incidence of acquired TMA is 17.5/million/year, 17% occur during pregnancy or postpartum [5]. aHUS has defects in complement regulation causing increased alternative pathway activation in glomerular vessels. The causes are disabling mutations in the genes of complement factor H (*CFH*), membrane cofactor protein (*MCP*; *CD46*), complement factor I (*CFI)* and thrombomodulin (*THBD*), and enabling mutations in factor B and C3 [6]. Autoantibodies against *CFH* sometimes occur [7]. Dense deposit disease (formerly membranoproliferative glomerulonephritis type II) and C3 glomerulonephritis are also associated with uncontrolled complement activation and mutations in *CFH* or *CFI* have been described [8]. The same mutations have occasionally been found in both C3 glomerulonephritis and aHUS [9]. This, in combination with incomplete penetrance of aHUS, indicates that other genetic or environmental factors trigger these diseases.

Anti-GBMGN has been associated with TMA (in particular TTP) and immune complex glomerulonephritis. Both diseases were diagnosed in the same person, typically with TMA diagnosed shortly after anti-GBMGN debut [10,11]. To our knowledge anti-GBMGN has never been associated with aHUS and anti-GBMGN and TMA has never been described within families. We present a daughter and her biological mother diagnosed with pregnancy-induced TMA and anti-GBMGN respectively, with 14 years in between and describes our exploration of a suspected link between these two rare diseases.

## Case presentation

### Daughter

A 22-year-old female was admitted with acute kidney failure following abruptio placentae causing severe vaginal haemorrhage in gestation week 33, resulting in stillbirth. The patient had a history of abortions in gestation weeks 9 and 28. Placental infarction at the second miscarriage resulted in heparin treatment during the actual pregnancy, which had been normotensive without proteinuria 2 days before admission.

**Figure 1:**
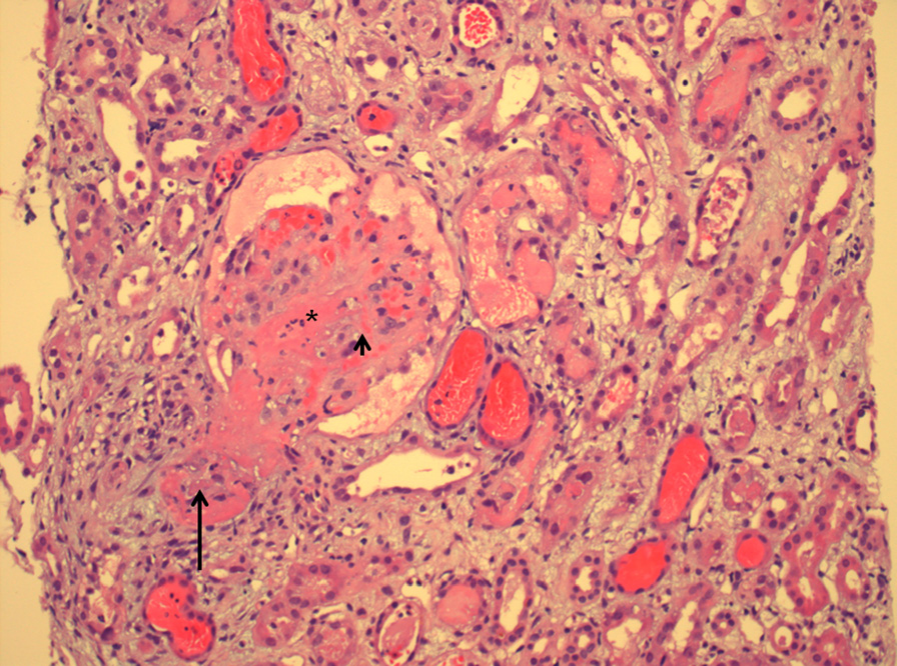
**TMA/aHUS. **Light microscopy, x200, hematoxylin and eosin stain. Thrombosis of the afferent arteriole (**↑**, large arrow) and partial necrosis of the glomerulus with deposition of fibrin (*) and fragmented erythrocytes (**↑**, small arrow).

She presented with haemolytic anaemia, thrombocytopenia, schistocytes, uraemia, hypertension, oliguria and proteinuria (Table 1). Treatment included plasmapheresis, haemodialysis and glucocorticoids. Anti-cardiolipin immunoglobulins, anti-Scl-70 and anti-double stranded DNA were negative. Anti-GBM and ANCA titers were not examined at disease onset, but were negative when examined 14 years later. Renal biopsy showed necrotic glomeruli with thrombi, platelet deposits and fibrin, vascular changes with luminal narrowing and intimal thickening, but no deposits. The diagnosis was TMA (pathologist Thomas Horn, MD, DMSc, Herlev Hospital, Denmark) (Figure 1). A grand mal seizure occurred during hospitalization (no simultaneous metabolic derangements or severe hypertension). The clinical diagnosis was pregnancy-induced TMA with components of aHUS and TTP. After 6 months, kidney function improved and the patient was temporarily dialysis-independent for three years. She was never transplanted.

**Table 1:** Baseline data

**Compound and Unit – SI units**	**Reference**	**Daughter, (TMA/aHUS)**	**Mother, (anti-GBMGN)**
Haemoglobin, mmol/L (g/L)	7.0–10.0 (113–161)	5.3 (85)	6.1 (98)
Platelets, ×10^9^/L	150–400	46	533
Haptoglobin, μmol/L	4–23	<5	13
Lactate dehydrogenase, U/L	150–450	7724	313
Bilirubin, μmol/L	4–17	36	9
Peripheral smear		>5% Schistocytes (Coombs test not performed)	n/a
White blood cell count × 10^9^/L	3.0–9.0	18.3	21.0
C-reactive protein, nmol/L	<95	n/a	981
Activated partial thromboplastin time, seconds	23–35	49	26
Factor II + VII + X, U/L	>0.60	1.00	0.85
Creatinine, μmol/L	40–110	430	799
Urea nitrogen, mmol/L	2.5–7.5	15.0	30.7
Potassium, mmol/L	3.5–5.0	6.6	5.9
Sodium, mmol/L	136–146	137	131
Phosphorus (inorganic), mmol/L	0,80–1,50	1,99	2,37
Calcium, mmol/L	2,20–2,60	2,18	2,15
Albumin, g/L	36–48	28	37
Bicarbonate, mmol/L	23–31	20	21

Baseline data at admission. Reference interval according to Danish standards.

### Mother

**Figure 2:**
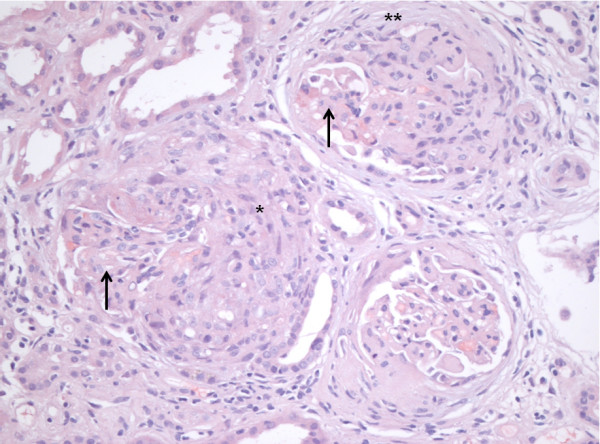
**anti-GBMGN.** Light microscopy, x200, hematoxylin and eosin stain. Diffuse extracapillary glomerulonephritis with predominant fresh crescentic formations (*) and a few older cresentic formations with fibrosis (**). Compression of the preserved part of glomeruli (**↑**).

A 69-year-old female was admitted with anuria and acute kidney failure. The patient had a 6-year hypertension history, hypothyroidism for 30 years and a minor stroke 4 months earlier treated by carotid thrombendarterectomy. At that time p-creatinine was normal. Blood tests showed severe uraemia and anaemia, but no haemolysis (Table 1). The anti-GBM titer was positive, 95 U/mL (ELISA-kit, Wieslab, Sweden; ELISA-reader TECAN, Switzerland) and myeloperoxidase anti-neutrophil cytoplasmic antibodies titer (MPO ANCA) was 25 U/mL. Proteinase-3 ANCA was negative (ELISA-reader TECAN, Switzerland) and chest X-ray normal. Treatment included methylprednisolone, cyclophosphamide, plasmapheresis and haemodialysis. Renal biopsy showed diffuse extracapillary glomerulonephritis with predominantly fresh crescentic formations, focal and segmental necrosis and linear deposition of IgG along the glomerular basement membrane, consistent with anti-GBMGN (pathologist Claus B. Andersen MD, DMSc, Rigshospitalet, Denmark) (Figure 2). The anti-GBM and MPO ANCA titres normalised after 16 days. There were no signs of TMA at any time. Kidney function was not regained.

## Additional analyses

Following maternal diagnosis, additional information was obtained and the following analyses were done:

*Family history:* Further elaboration of the family history of kidney disease was done in order to identify additional cases. No other family members had ever experienced clinically significant kidney diseases or any signs of kidney disease. It was not possible to obtain blood samples for genetic analyses from the father. Mother and daughter lived in the same household until a few years before disease onset of the daughter. There were no obvious exposures to environmental factors such as drugs, hydrocarbons or other toxins. They discharged different occupations.

*HLA tissue typing* (Dynabeads, Dynal, Norway): *HLA-A1,26;B7,8;DR2,3* (mother), *HLA-A1;B8,37;Cw6;DR3,6* (daughter). Subtyping was not performed.

*ADAMTS13 activity and antibodies* (CytoFlour® 4000 Fluorescence Plate Reader, Applied Biosystems Inc, USA): ADAMTS13-protein 0.61 U/L (0.75-1.33) (mother); 0.97 U/L (daughter). No antibodies.

*The complement system* (Wielisa, Wieslab, Sweden): Both patients had normal alternative- and classical pathway function. The daughter had reduced Mannose-binding lectin (MBL) pathway activity, and additional gene typing (SSP-PCR) revealed gene type XA/B, compatible with a severely reduced MBL level*.* Gene sequencing of *CFH, CFI, MCP* and *THBD* showed heterozygosity for a *CFI* mutation (G261D, alternative syntax G243D) and several disease risk associated polymorphisms in the daughter. The mother was non-carrier of the G261D mutation but shared the disease associated *CFH* Q672Q silent polymorphism (Table 2). *CFH*-autoantibodies were not investigated, but none of the patients were carriers of the common *CFHR1/CFHR3* deletion or other *CFHR1* deletions strongly associated with antibody induced aHUS [12]. Measurement of C3 levels were not performed.

**Table 2:** Genetic analyses

**Gene**	**Variation**	**Description**	**Disease association**	**Daughter, (TMA/aHUS)**	**Mother (anti-GBMGN)**
***CFI***	NG_007569				
	c.5256A>G	Intron rs7671905	Polymorphism	homozygous	homozygous
	c.42455C>T	Intron rs79375065	Polymorphism	heterozygous	Non-carrier
	c.46524G>A	Intron rs4382037	Polymorphism	homozygous	homozygous
	**c.46615G>A**	**G261D**	Mutation reported in MPGN and aHUS	heterozygous	Non-carrier
	c.49140C>A	Intron rs7437142	Polymorphism	homozygous	homozygous
	c.49159ins AATTT	Intron rs78629056	Polymorphism	homozygous	homozygous
	c.57335C>G	Intron rs7441380	Polymorphism	homozygous	homozygous
	c.66205C>T	Intron rs551	Polymorphism	homozygous	homozygous
***CFH***	NG_007259.1				
	c.38184A>C	A307A	Disease risk polymophism (AMD)	heterozygous	Non-carrier
	c.43097C>T	H402Y	Disease risk polymophism (AMD)	heterozygous	Non-carrier
	c.79602A>G	Q672Q	Disease risk polymorphism (aHUS)	heterozygous	heterozygous
	c.89786C>A	Intron rs375046	Polymorphism	heterozygous	heterozygous
	c.93634G>T	E936D	Disease risk polymorphism (aHUS)	heterozygous	Non-carrier
***MCP***	NG_009296				
	c.12610A>G	L139L rs12126088	Rare non-Disease Causing Polymorphism	heterozygous	Non-carrier
	c.20790G>T	Intron rs2724374	Polymorphism	heterozygous	Non-carrier
	c.36158G>A	Intron rs1962149	Polymorphism	heterozygous	Non-carrier
***THBD***	NG_012027				
	c.6578C>T	A473V rs1042579	Polymorphism	homozygous	heterozygous

Genetic screening for variation in the *CFI-, CFH-, MCP-* (*CD46*) and *THBD*-genes. Position of the variations in the respective GeneBank refrence sequences as well as dbSNP numbers are listed.

*CFI* = Complement Factor I, *CFH* = Complement Factor H, *MCP* = Membrane Cofactor Protein, *THBD* = Thrombomodulin, *AMD* = Age-related Macula Degeneration.

## Conclusions

The occurrence of two very rare renal diseases within the same family is likely to be explained by a common aetiology. One linkage could be a common autoantibody-profile; however this was not demonstrated. ANCA-positive testing in anti-GBMGN occurs in 30% [13] and HLA tissue typing demonstrated known predisposing relations without any conspicuous shared patterns. The mother’s slightly reduced ADAMTS13-protein is probably not significant. However, the daughter’s normal ADAMTS13 activity and antibody absence during screening, does not exclude affection at diagnosis. The daughter’s G261D *CFI*-gene mutation combined with disease-associated polymorphisms may predispose to the pregnancy-induced TMA. The G261D mutation has been found in other patients with C3 glomerulonephritis and aHUS [9,14]. Despite several tests by Nilsson *et al.,* no functional effect of the G261D mutation on *CFI* mediated complement regulation or on *CFI* serum levels could be demonstrated [14]; however, it may have effects not revealed by the in vitro tests, or be a marker for a linked genetic deficiency. The silent G672G polymorphism in both patients is strongly associated with aHUS [15], and even though this is unlikely to be *the* shared aetiology, it is possible that it influenced the disease in both patients. Reduced MBL activity was demonstrated in the daughter but not the mother. This has no known relation to TMA (or anti-GBMGN).

In conclusion, we have found a shared *CFH* polymorphism that may confer increased complement-mediated disease risk, but no other connection between TMA and anti-GBMGN. The diseases may require multiple triggers including mutations, polymorphisms, autoantibodies and perhaps infections, and a common genetic susceptibility cannot be ruled out.

## Consent

Written informed consent was obtained from both patients for publication of this case report and accompanying images of the kidney biopsies. Acceptance was noted in the patients’ records. A copy of the written consent is available for review by the Editor-in Chief of BMC Nephrology.

## Abbreviations

anti-GBMGN, Anti-glomerular basement membrane glomerulonephritis; TMA, Thrombotic microangiopathy; TTP, Thrombotic thrombocytopenic purpura; aHUS, Atypical HUS; CFH, Complement factor H; CFI, Complement factor I; MCP, Membrane cofactor protein; THBD, Thrombomodulin.

## Competing interests

The authors have no financial or non-financial competing interests and no disclosures.

## Authors’ contributions

TI: Participated in research design, collection of data and blood samples, information of the patients, data analysis and writing of the paper. LS: Participated in research design, gene analyses, data analysis and writing of the paper. CR: Participated in collection of data and blood samples, information of the patients and writing of the paper. JGH: Participated in research design, information of the patients, data analysis and writing of the paper. KRJ: Participated in gene analyses and writing of the paper. MB: Participated in gene analyses and writing of the paper. PG: Participated in research design, gene analyses, data analysis and writing of the paper. ALK: Participated in research design, data analysis and writing of the paper. All authors have read and approved the final version of the article.

## Pre-publication history

The pre-publication history for this paper can be accessed here:

http://www.biomedcentral.com/1471-2369/13/64/prepub
